# Specific inter-domain interactions stabilize a compact HIV-1 Gag conformation

**DOI:** 10.1371/journal.pone.0221256

**Published:** 2019-08-22

**Authors:** Chen Lin, Paola Mendoza-Espinosa, Ioulia Rouzina, Orlando Guzmán, José Antonio Moreno-Razo, Joseph S. Francisco, Robijn Bruinsma

**Affiliations:** 1 Department of Chemistry and Biochemistry, University of California Los Angeles, Los Angeles, CA, United States of America; 2 Departamento de Física, Universidad Autónoma Metropolitana, Iztapalapa, Ciudad de México, México; 3 Department of Chemistry and Biochemistry, The Ohio State University, Columbus, OH, United States of America; 4 Department of Chemistry, The University of Pennsylvania, Philadelphia, PA, United States of America; 5 Department of Physics and Astronomy, University of California Los Angeles, Los Angeles, CA, United States of America; University of Colorado Denver Skaggs School of Pharmacy and Pharmaceutical Sciences, UNITED STATES

## Abstract

HIV-1 Gag is a large multidomain poly-protein with flexible unstructured linkers connecting its globular subdomains. It is compact when in solution but assumes an extended conformation when assembled within the immature HIV-1 virion. Here, we use molecular dynamics (MD) simulations to quantitatively characterize the intra-domain interactions of HIV-1 Gag. We find that the matrix (MA) domain and the C-terminal subdomain CA_ctd_ of the CA capsid domain can form a bound state. The bound state, which is held together primarily by interactions between complementary charged and polar residues, stabilizes the compact state of HIV-1 Gag. We calculate the depth of the attractive free energy potential between the MA/ CA_ctd_ sites and find it to be about three times larger than the dimerization interaction between the CA_ctd_ domains. Sequence analysis shows high conservation within the newly-found intra-Gag MA/CA_ctd_ binding site, as well as its spatial proximity to other well known elements of Gag –such as CA_ctd_’s SP1 helix region, its inositol hexaphosphate (IP6) binding site and major homology region (MHR), as well as the MA trimerization site. Our results point to a high, but yet undetermined, functional significance of the intra-Gag binding site. Recent biophysical experiments that address the binding specificity of Gag are interpreted in the context of the MA/CA_ctd_ bound state, suggesting an important role in selective packaging of genomic RNA by Gag.

## Introduction

Despite intense research efforts, important aspects of the life-cycle of the HIV-1 virus are not understood. One of these unresolved issues concerns the initiation of the assembly of the capsid of HIV-1 virus particles (also known as virions) [1]. This capsid is a shell composed by some 2400 to 5000 molecules of the large viral Gag polyprotein (**G**roup-specific **a**nti**g**en) [2]. It encloses the viral genome composed of two copies of the single-stranded viral genomic RNA (gRNA), each about 10 kilobase long [3]. During assembly, the gRNA molecules must be selected from among an overwhelming majority of host cytoplasmic RNA. Gag is the only protein required for the production of virus-like particles (VLPs): Gag expression in transfected cells lacking gRNA leads to release of non-infectious VLPs that are morphologically indistinguishable from infectious viruses and have the same total amount of RNA packaged. However, instead of gRNA, these VLPs package host RNA molecules in proportion to their cytoplasmic presence [1].

The unresolved issue in this context concerns the gRNA selection mechanism: despite intense studies in cells and *in vitro* by many labs, HIV-1 Gag proteins appear to have hardly any binding specificity for gRNA over generic RNA; yet, over 90 percent of the virions produced in the infected cell do carry HIV-1 gRNA molecules [4, 5]. Current evidence suggests that gRNA selection takes place during the first capsid assembly steps [1], which involve few Gag molecules binding gRNA in the cytoplasm [6, 7]: It appears that under conditions typical of the virion assembly corresponding to the low cytoplasmic levels of Gag, only the Gag molecules bound to gRNA can attach to the plasma membrane (PM) and initiate the assembly. In contrast, the majority of Gag bound to non-viral RNA remains monomeric in the cytoplasm and is incapable of assembly nucleation [1]. According to this view, selection happens at the step where the first Gag molecules bind to the *Ψ* recognition element of gRNA in the cytoplasm [8, 9] and, therefore, must be a feature of individual or a small number of interacting Gag proteins.

**Fig 1:**
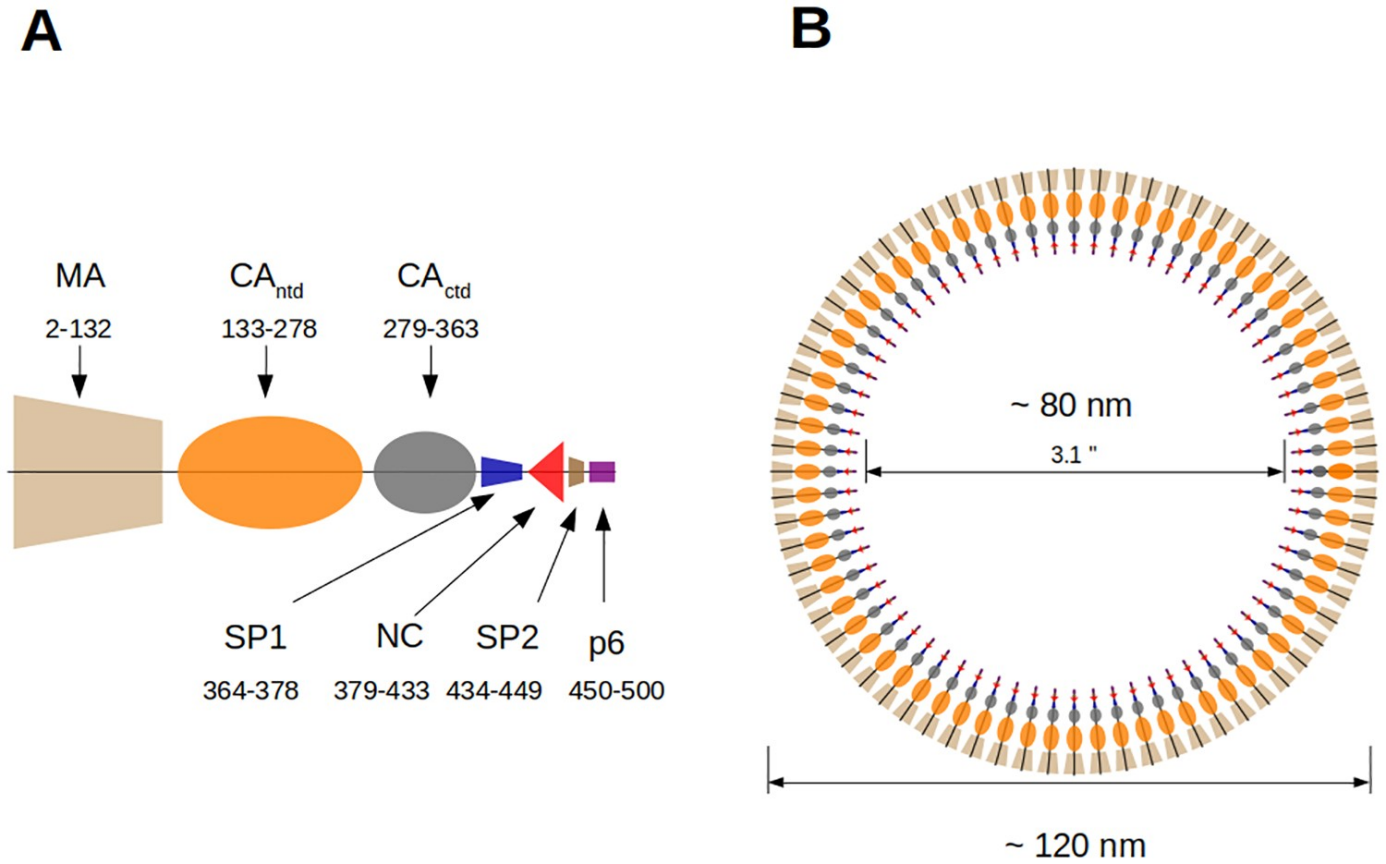
Schematics of Gag domains and HIV-1 immature capsid. A: The MA domain of Gag has a large number of positively charged residues (at neutral pH) while the CA domain, which is composed of the linked CA_ntd_ and CA_ctd_ subdomains, is close to neutral. The NC (nucleocapsid) domain, which is separated from the CA_ctd_ subdomain by the short SP1 sequence, has a net positive charge. Spacer SP2 sits between NC and the unstructured peptide p6. B: In the HIV-1 immature capsid, the positively charged MA domains of the Gags are associated with the negatively charged plasma membrane (not shown) on the exterior of the capsid. The CA_ctd_ domains of adjacent Gags are bonded by hydrophobic interactions while positively charged NC domains are associated with the negatively charged viral RNA molecules (not shown) in the capsid interior.

As shown in Fig 1, the Gag polyprotein is composed of a number of domains that are conventionally labelled as MA, CA_ntd_, CA_ctd_, NC and p6. MA stands for “**m**embrane **a**ssociated”, CA stands for “**ca**psid”, ntd and ctd refer to the N and C terminal domains of CA, NC stands for “**n**ucleo-**c**apsid”). The highly-variable, unstructured peptide p6 plays a role in virion budding, while recent experiments also point to a possible contribution to selective gRNA encapsidation [10]. All Gag domains are connected by flexible linkers. The longest one, between MA and CA, consists of 30 unstructured amino acids (aa) and is the major element of Gag flexibility, while the linker between CA_ntd_ and CA_ctd_ is only 4 aa long. The SP1 region between CA_ctd_ and NC is unstructured in monomeric Gag but has propensity for *α*-helix formation both in low dielectric media and upon Gag oligomerization [11]. In addition, interactions among SP1 regions of adjacent Gag molecules have been shown to contribute significantly to the inter-Gag interactions that stabilize the immature capsid lattice [12–14]. Finally, the SP2 peptide joins NC to p6.

In the immature virion, the Gag proteins form a hexagonal lattice [14, 15] stabilized primarily by hydrophobic interactions between CA_ctd_ domains and SP1 six-helix bundle [13–16]. A single-molecule FRET (Förster resonance energy transfer) study of Gag proteins in solution [17] reported that they have a range of conformations, some with the MA and NC domains in close proximity –to be denoted as “compact Gag” or “C-Gag”– and some with these two domains much further (beyond the 8 nm Förster radius) –to be denoted as “extended Gag” or “E-Gag”. The E-Gag state has a broad MA-NC distance probability distribution with relatively large MA-NC distances, suggesting that the E-Gag state has significant conformational fluctuations. In contrast, the C-Gag state has a narrower MA-NC distance distribution with relatively short (within 8 nm) MA-NC distances. The C-Gag and E-Gag states seem to be stable and do not interconvert over typical FRET observation times (up to 100 s). Importantly, the compact state is the majority component of monomeric Gag in solution and becomes even more abundant at higher Gag concentrations, or upon addition of single-strand DNA polyA oligonucleotides. Following up on that finding, in a recent MD simulation of Gag [18], the different domains of Gag were found to move almost independently as rigid bodies linked by flexible tethers if the initial state corresponded to the extended Gag state of the FRET experiments. The authors also identified another state, with the MA and CA_ctd_ domains in close contact with each other, starting from a configuration derived from the FRET constraint to the MA-NC distance. In this state, which could correspond to the compact state, the MA and CA_ctd_ domains are quite correlated, indicative of long-range allosteric interactions [19]. Both states remained stable over a simulation time of 300 ns.

The fact that the majority component of Gag in solution is in the C-Gag conformation suggests a possible gRNA selection mechanism. Assume that the observed lack of affinity of Gag proteins for gRNA is not a property of Gag in general but specific only for C-Gag, as suggested by *in vitro* binding studies [20, 21]. If E-Gag would have increased binding specificity for gRNA (due to allosteric coupling) and an increased ability to associate with other Gag molecules (due to exposed sites of CA/CA interaction) then the C-to-E transition could act as a regulatory assembly gateway that prevents or retards capsid assembly on non-specific RNAs. In this paper, we report on MD simulations of the interactions between individual domains of Gag. Based on the earlier simulation study [18], we first hypothesized that the physical mechanism that stabilizes the C-Gag state involves strong attractive intra-molecular interactions between the MA and CA_ctd_ domains. Next, the CA_ctd_ domain has exposed hydrophobic residues on its outer surface (W316, M317 residues referred as WM dimerization site) and homo-dimeric hydrophobic interactions between adjacent, charge-neutral CA_ctd_ domains are known to make an important contribution to the stabilization of assembled hexagonal arrays of Gag proteins. For this reason, we initially hypothesized that the WM dimerization site of CA_ctd_ also stabilizes the intra-molecular MA/CA_ctd_ bound state, providing an effective way to prevent assembly of Gag proteins. The all-atom molecular dynamics (MD) simulations discussed below on MA/CA_ctd_ interaction and on CA_ctd_ WM-dimeric interaction support the first but not the second hypothesis: we found a novel MA/CA_ctd_ binding site different from the WM dimerization one, of predominantly electrostatic character, and that such intra-Gag interaction is significantly stronger than the WM-mediated CA_ctd_ dimerization.

## Methods

### Selection of candidate MA/CA_ctd_ binding and CA_ctd_/CA_ctd_ dimer configurations

Atomic coordinates of MA and CA_ctd_ were obtained from Protein Data Bank (PDB) files (PDB ID 2H3F for MA [22] and PDB ID 4IPY for CA_ctd_ [23]). For the MA and CA_ctd_ pair, we produced initial candidate geometries by applying the PyDock program [24] which ranks the possible binding configurations of the complex taking into account electrostatic, van der Waals, and desolvation interaction. The top four ranked configurations that were not precluded from steric impediments with other components of Gag were examined in 25 ns MD simulations using the GROMACS 4.6.5 software package [25]. We applied the GROMOS96 53A6 force field to model the proteins [26] and three different models of water: SPC/E [27], TIP3P [28] and TIP4P*ϵ* [29]; we repeated the simulation of the top four ranked configurations using the CHARMM36 force field for the proteins [30] and TIP3P water for 30 ns. The only MA/CA_ctd_ pairing state that remained together in their initial configuration over the GROMOS96 and CHARMM36 simulations was selected as the initial geometry for protein-protein interaction analysis and umbrella sampling calculations of potential of mean force (see below).

For the CA_ctd_/CA_ctd_ dimer, we applied GROMOS96 53A6 and SPC/E as the force fields and used the NMR structure from PDB 4USN (measured to a resolution of 8.8 Å) [15], as this corresponds to the configuration in the immature-virion lattice. We checked that this structure for CA_ctd_ matches that provided later by the same and other groups (see Supporting Information S1 Appendix and S1 Fig): in the first case the root-mean-square deviation of atomic positions for CA_ctd_ between structures 4USN and 5L93a [14] is 1.45 Å, which is less than the 3.9 Å resolution of the 5L93a structure. Regarding comparisons with structures determined by other groups, the root-mean-square deviation between CA_ctd_ structures from 4USN and 5I4Tg [31] is 1.27 Å, and that between 4USN and 6N3Ja [32] is 1.41 Å. The resolutions to which structures 5I4T and 6N3J were measured are 3.27 Å and 2.9 Å, respectively.

For each pair MA/CA_ctd_ and CA_ctd_/CA_ctd_, the N-terminus of terminal peptide groups were capped with an acetyl group in order to produce an uncharged terminus, while the C-terminus of each peptide was deprotonated. Short-range non-bonded interactions were cut off at 1.4 nm, while long-range electrostatic interactions were obtained by the Particle Mesh Ewald (PME) algorithm [33, 34]. This was facilitated by applying periodic boundary conditions in all directions.

### Protein-protein interaction study of the MA/CA_ctd_ binding interface

The MA/CA_ctd_ binding interface study was undertaken using the following freely-accesible online servers, each providing a protein-protein interaction (PPI) analysis algorithm: SPPIDER [35], InterProSurf [36], PIC [37], BindProfX [38], COCOMAPS [39], PDBePISA [40] and KFC2 [41]. Structure visualization was performed with VMD [42] and UCSF Chimera [43].

As input to each of the PPI algorithms, we provided the bound MA/CA_ctd_ structure obtained from pydock and confirmed to be bound after 25 ns of MD simulation; such structure was supplemented with the missing hydrogen atoms in the source PDB files. Each algorithm produced as output a list of candidate amino acids belonging to the binding interface, which we provide in S2 Appendix in the Supplementary Information. In this work, we focus only on those aa that were listed by all seven PPI algorithms, but also consider that other contacts out of this consensus may play a role. To explore this issue, we performed additional 50 ns-long NPT molecular dynamics simulations with the consensus aa replaced progresively with alanines: we analyzed the shifts in the distribution of distances between residues 43 and 344 (two complementary-charged consensus residues in the wild-type MA and CA_ctd_ domains).

The PDBePISA PPI analysis indicated that the solvent-accessible surface area was 5565.4 Å^2^, while that of the interface was 510.6 Å^2^. From the same source, we obtained a solvation free energy Δ^*i*^*G* = −4.7 kcal/mol once the interface is formed. The negative value indicates affinity between MA and CA_ctd_, but this value does not include the effect of formation of hydrogen and salt bonds at the interface.

### Umbrella sampling calculation of potential of mean force

In order to measure the potential of mean force (PMF) with respect to the center of mass (COM) separation *X* of the MA/CA_ctd_ and CA_ctd_/CA_ctd_ complexes, we used the umbrella sampling method [44] as implemented in GROMACS (see S2 Appendix). For this analysis we only used the combination of the GROMOS96 53A6 force field [26] and SPC/E water [27]. For the first pair, the MA domain was immobilized using the PDB coordinates with the center of mass fixed at the origin, while the CA_ctd_ group was unconstrained; in the second pair, one CA_ctd_ group was immobilized and the second one left unconstrained.

Each complex was simulated in a rectangular box with periodic boundary conditions, with a size of 10 × 11 × 18 nm^3^ for MA/CA_ctd_ and 10 × 11 × 16 nm^3^ for CA_ctd_/CA_ctd_. Each box was filled by SPC/E water, while 0.1 M NaCl was added to allow for electrostatic screening. Before generating the set of umbrella-sampling windows with a pulling protocol, we first equilibrated each complex. Then, the center of mass of the mobile CA_ctd_ group was exposed to a rotationally symmetric parabolic umbrella potential. Shifting the equilibrium point of this harmonic potential, the mobile CA_ctd_ group was pulled away from the immobile group.

The pulling process was performed along the projection of center of mass separation on the longest axis of the simulation box. A spring constant of 1300 kJ/mol nm^2^ and a 0.01 nm/ps pull rate were used for both systems, over a time of 700 and 600 ps for MA/CA_ctd_ and CA_ctd_/CA_ctd_, respectively. From the pulling-process simulation trajectories, 16 snapshot configurations were selected as starting states for the umbrella sampling of MA/CA_ctd_, and 13 for CA_ctd_/CA_ctd_. The separation between the starting states and the stiffness of the umbrella potential guaranteed that there was enough overlap between different sampling windows.

In each window, a 100 ps NPT equilibration run was performed first. Next, a 12 ns constrained MD simulation with umbrella spring constant 2000 kJ/mol nm^2^ was run to collect the sample. Analysis of results was carried out by the weighted histogram analysis method (WHAM) [45]. The spring constant of the parabolic umbrella potential was chosen to be sufficiently strong so the equilibrium probability distribution of the COM coordinate of the CA group was close to a Gaussian. As a a check of the procedure, the force exerted by the PMF at each location was directly obtained from the shift of the centroid of the Gaussians from the origin of their respective umbrella potentials, with results consistent with the PMF obtained from WHAM.

## Results

### Interactions between the MA and CA_ctd_ domains

**Fig 2:**
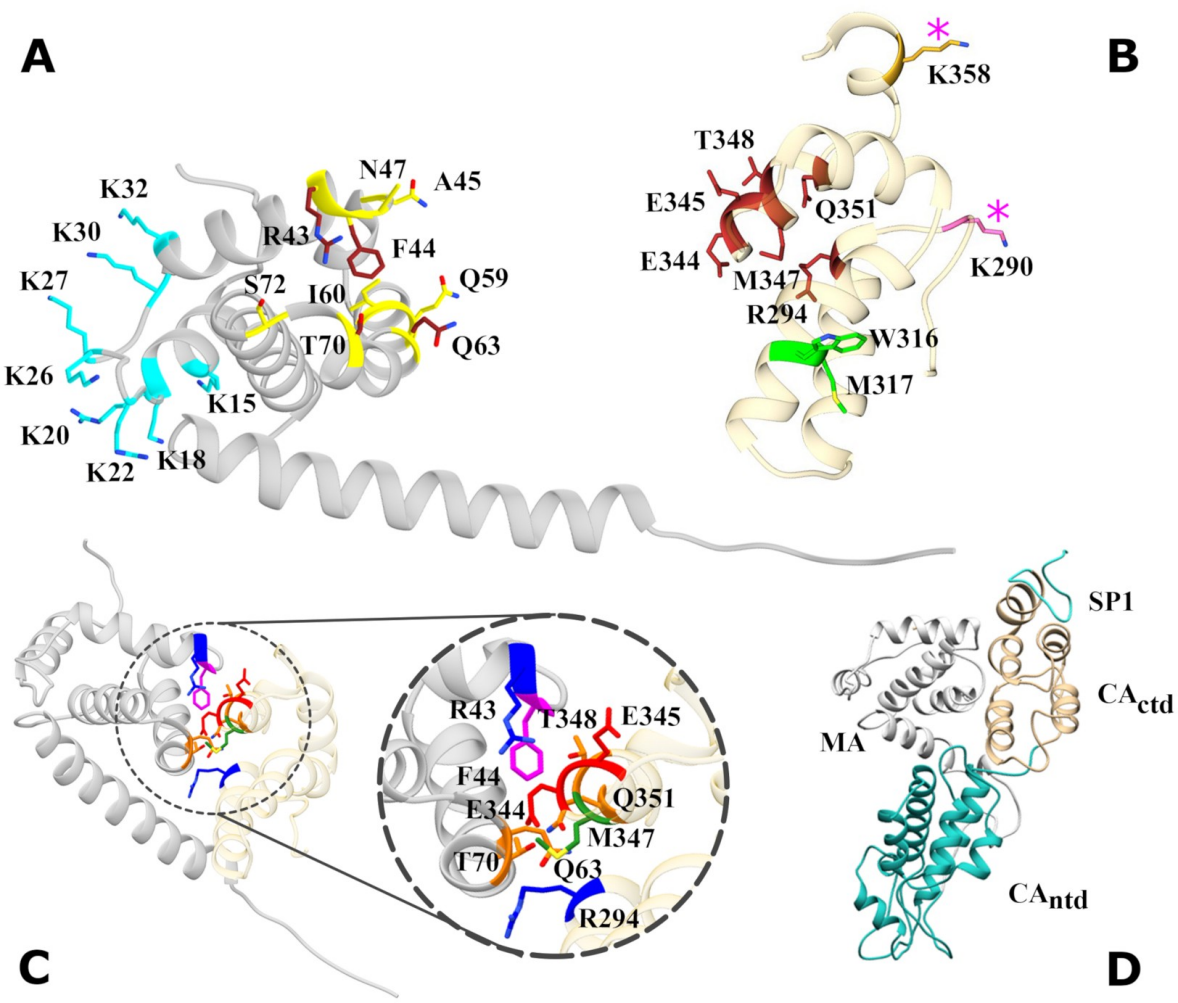
Structure of MA, CA_ctd_ and MA/CA_ctd_ bound state. A: The entire MA domain is shown in gray. The annotated residues belong to the highly basic region HBR (cyan), the MA-MA trimerization site (yellow) and the newly found, intra-Gag MA/CA_ctd_ binding site (red). B: the CA_ctd_ subdomain is shown in tan. Annotated residues belong to the two-fold CA_ctd_/CA_ctd_ (green) and six-fold CA_ctd_/CA_ctd_ (orange) interaction sites in the immature capsid, the inositol hexaphosphate (IP6) binding site (pink asterisks), and the MA/CA_ctd_ intra-Gag binding site (red). C: The MA/CA_ctd_ bound state, with participating residues colored by their character: positively charged (blue), negativley charged (red), polar (gold) and nonpolar (violet). D: Snapshot of the MA/CA_ctd_ bound state from a molecular dynamics simulation of Gag including MA (gray), CA_ntd_ (blue), CA_ctd_ (tan), and SP1 (cyan) in explicit water (not shown).

In order to search for an MA/CA_ctd_ bound state, we constructed a list of possible MA/CA_ctd_ pairing configurations arranged in terms of binding scores computed using standard protein-protein docking software as described in the Methods section. Next, each candidate configuration underwent short MD simulations (25 ns and 30 ns, respectively when using GROMOS96 and CHARMM36). Only one of the candidate configurations, the one shown in Fig 2C, survived bound throughout simulations with all combinations of the protein and water force fields tested. Simulations for the other three candidates show that they soon loose their original contacts: MA and CA_ctd_ tend to roll over their surface, losing their initial relative orientation before their centers of mass separate.

For the bound configuration in Fig 2C, as described in the Methods section, we focus on those residues identified by consensus of all seven protein-protein interaction algorithms used (the full lists output by each PPI algorithm are given in S1 Table in Supplementary Information). These interacting residues in MA and CA_ctd_, as well as their position within each domain, are shown in Fig 2. In MA, these residues at the binding interface were found to be R43, F44, Q63 and T70; in CA_ctd_ they were R294, E344, E345, M347, T348, and Q351. While sites in both domains are overall neutral, electrostatic interactions dominate their binding interface. Specifically, we identified interaction between charged residues R43 (positive) and E344 and E345 (negative), between polar residues Q63, R294 and Q351, and between polar residue T70 and negatively charged E344. The only hydrophobic residues identified (by consensus of the PPI analysis) at the binding interface were F44 and M347.

**Fig 3:**
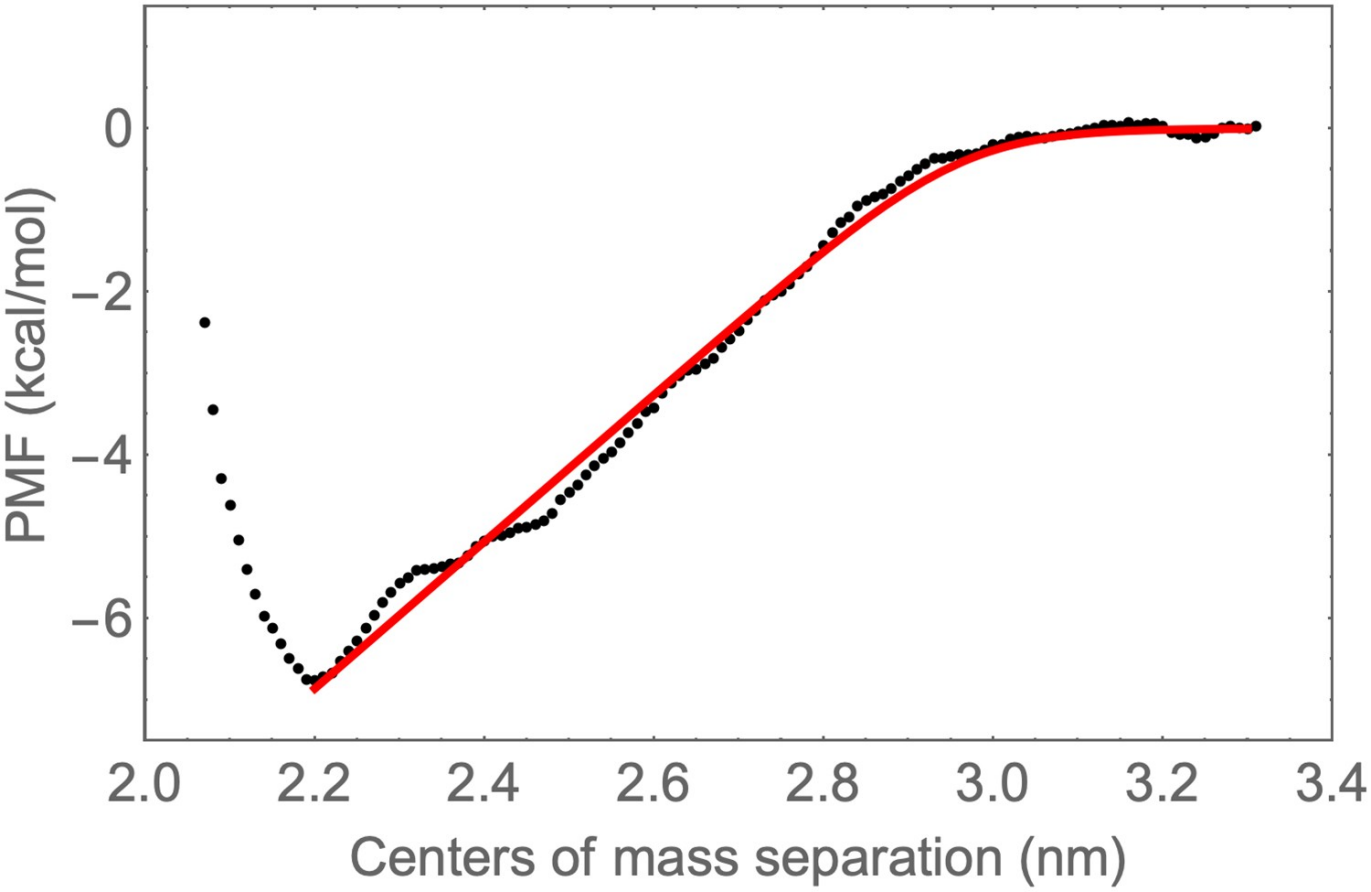
Potential of mean force for the MA/CA_ctd_ bound state. The PMF between the MA and CA_ctd_ subdomains obtained by umbrella sampling MD simulation (black points) is shown as a function of the distance *X* between their centers of mass. Red line: fit of the form *V*(*X*) = −*k*_*B*_*T* ln[1 + *e*^*β*(Δ*V* − *F*(*X* − *X*_0_))^], appropriate for a two-state mechano-chemical system.

To quantify the strength of the bound state, we computed the Potential of Mean Force (PMF) as a function of the MA/CA_ctd_ center of mass (COM) separation *X*, using the umbrella sampling method. The results are shown in Fig 3. The PMF has a potential well with a depth Δ*V*_*MC*_ of 6.8 kcal/mol (≃11*k*_*B*_T) while the COM separation *X*_0_ at the potential minimum is 2.2 nm. The PMF increases approximately linearly between 2.2 and 3.0 nm and levels off at longer distances. Polar interactions are the main contributors to the attraction in the linear regime of the MA/CA_ctd_ PMF. The linear part of the PMF translates into a constant attractive force *F* of about 44 pN. If the bound state is treated as a two-state mechano-chemical system subject to a constant force *F*_*i*_ then corresponding PMF would have the form *V*(*X*) = −*k*_*B*_*T* ln[1 + *e*^*β*(Δ*V* − *F*(*X* − *X*_0_))^], which fits very accurately the simulation results, as shown in Fig 3.

When the computation of the MA/CA_ctd_ PMF was repeated on approach, with an initial COM separation *X* increased to 3.2 nm but with the same relative orientation of the MA and CA_ctd_ domains as in the bound state, then the simulations failed to equilibrate. This was found to be due to rotational Brownian motion causing orientational misalignment between the MA and CA_ctd_ domains. The MA/CA_ctd_ interaction potential is apparently highly directional: the two domains need to be lined up precisely in order for a bond to form. The interaction appears to have a *ratchet-like* character with kinetic traps appearing on approach but not on separation.

**Fig 4:**
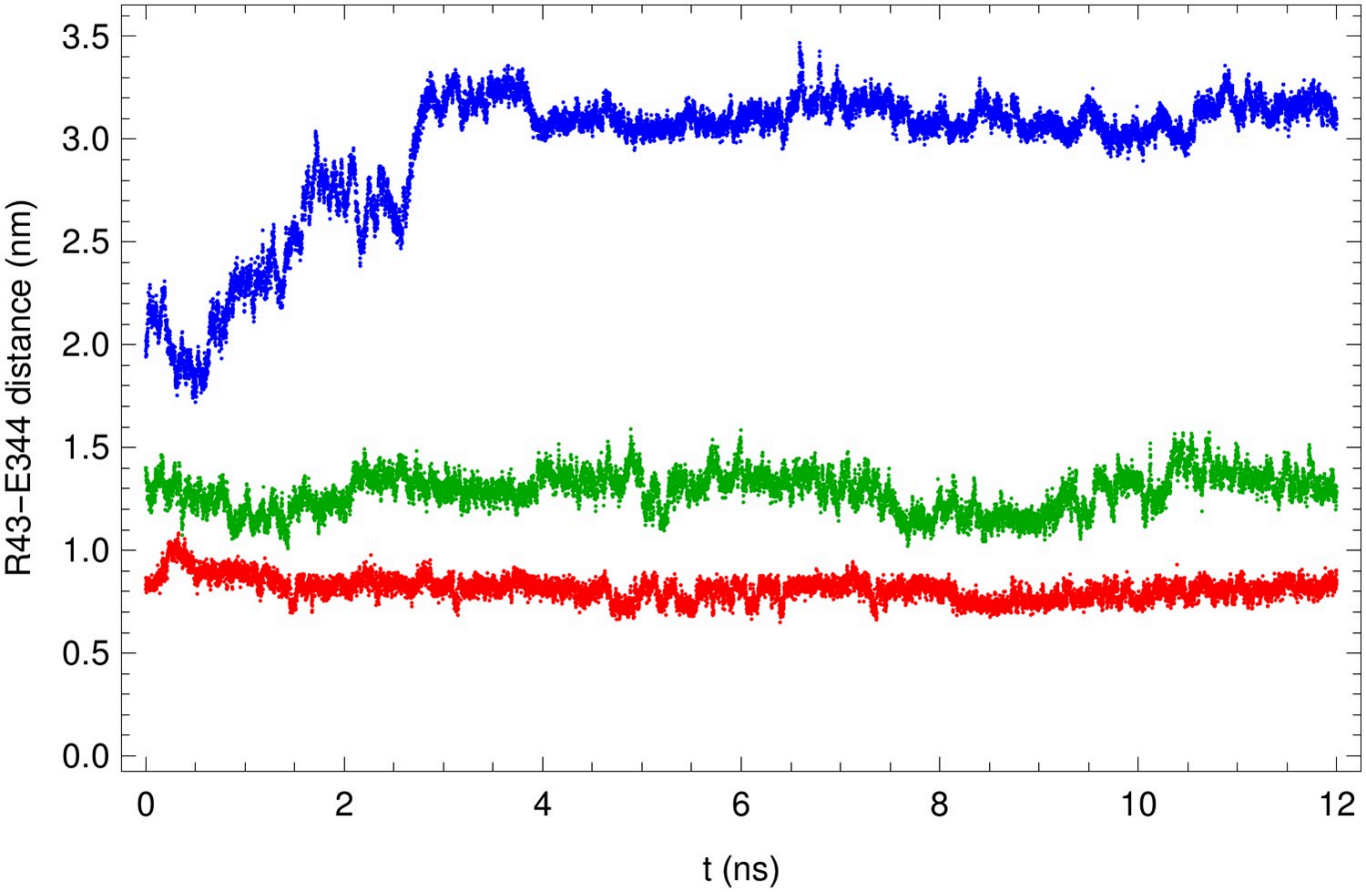
Analysis of electrostatic bond between MA and CA_ctd_. The separation between the COM of residues R43 of MA and E344 of CA_ctd_ is shown as a function of time for different values of the average COM separation of MA and CA_ctd_ domains: 〈*X*〉 = 2.3 nm (red), 2.7 nm (green), and 3.2 nm (blue). Contrary to the first two, the last case shows that the electrostatic bond between R43 and E344 snaps and gradually adopts a new equilibrium separation.

To gain insight into the effect of the COM displacement on the bonding between individual residues, we tracked the separation between the centers of mass of the positively charged R43 residue of MA and the negatively charged E344 residue of CA for different values of the mean COM separation〈*X*〉 of the two domains (See Fig 4). The COM of the MA domain was fixed while the COM of the CA domain was subject to a parabolic umbrella potential. For 〈*X*〉 = 2.3 nm, which is at the bottom of the PMF, the separation *s* between E43 and R344 fluctuates around a mean value of 0.81 nm with a standard deviation of 0.06 nm, the latter being comparable to the width of the applied umbrella potential. When the MA/CA_ctd_ separation 〈*X*〉 was increased to 2.7 nm, the separation *s* shows enhanced fluctuation around the mean value 1.29 nm with standard deviation 0.09 nm. The change in *s* is comparable to the increase of the COM separation 〈*X*〉, while the enhanced fluctuation is attributed to a combination of a rotation of the bonding direction and a structural deformation, but with the electrostatic bond apparently still intact. When the COM separation 〈*X*〉 was increased to 3.2 nm, the separation *s* increased steadily from its initial value of 1.97 nm until it finally equilibrated around a mean value of 3.11 nm with a standard deviation 0.07 nm. The natural interpretation in this case is that the electrostatic bond had snapped, which is consistent with the flat character of the MA/CA_ctd_ PMF at such a large value of 〈*X*〉.

**Fig 5:**
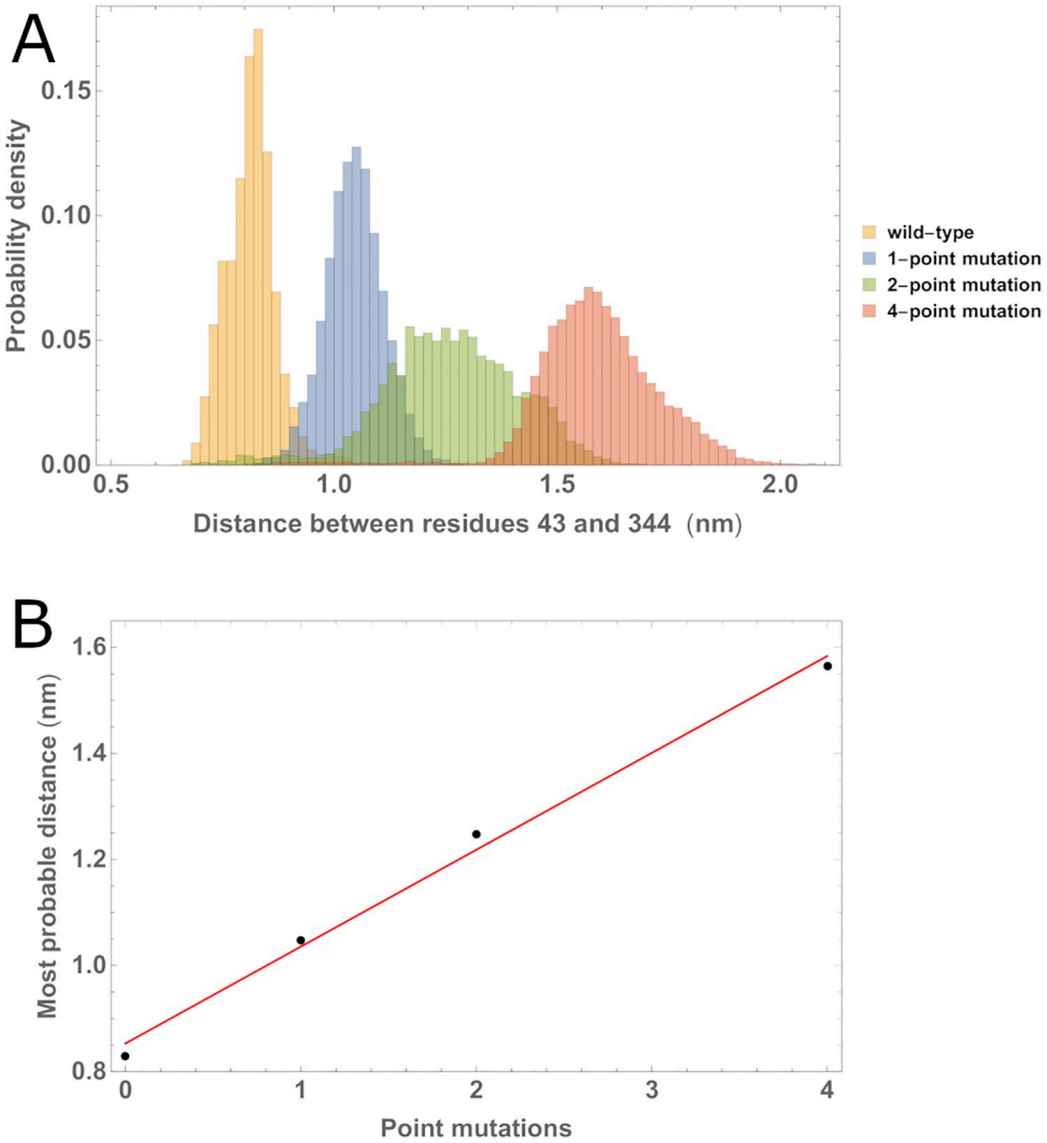
Effect of multiple alanine substitution of residues in the binding interface of MA. A: The distribution of separations between the COM of residues 43 of MA and 344 of CA_ctd_ shows a systematic shift toward larger values with increasing number of alanines replacing residues in the binding interface of MA: zero (yellow), one (blue), two (green) and four (pink). B: The most probable separation for these distributions (black dots) increases with the number of alanine point mutations and can be fitted with a linear model (red line).

In order to explore the effect of point-mutations to the residues identified by consensus of the PPI algorithms, we performed additional MD simulations where we replaced with alanines between 1 and 4 of the residues in the MA binding interface. We tracked the separation *s* between residues 43 and 344 for such multiple “point-mutation” MA/CA_ctd_ complexes over the entire duration of the simulations (50 ns). Fig 5 shows that the corresponding distributions of *s* present a systematic shift toward larger values as the number of alanine point mutations increses. Specifically, the most probable value of the separation *s* correlates strongly and increases linearly with the number of alanine point mutations. We interpret this as a gradual deterioration of the ability of MA to remain in close contact with CA_ctd_ as the number of wild-type contacts in its binding interface is reduced. Also, this indirectly shows that other residues in MA (beyond the four in the consensus list) have a role in holding CA_ctd_ at close distance. We come back to this point below in the Discussion.

Finally, because the focus was on the basic mechanism that stabilizes the compact state of Gag, the simulations did not include interactions of MA and CA_ctd_ with the CA_ntd_, SP1, and NC subdomains of Gag. It could be questioned whether the bound state will even survive when these interactions are included. To check for that, we carried out an explicit-water simulation of Gag including MA, CA_ntd_, CA_ctd_, SP1 and the linkers between them, using an initial state in which the MA/CA_ctd_ pair is bound (see Fig 2D). The intra-molecular bound state of Gag remained intact over the simulation. However, due to the very large size of this Gag model, the duration was limited to 50 ns. Below, we will comment on the additional interactions in terms of how they may modulate the strength of the bound state, which is a subject of future study.

### Homodimeric CA_ctd_ interactions

**Fig 6:**
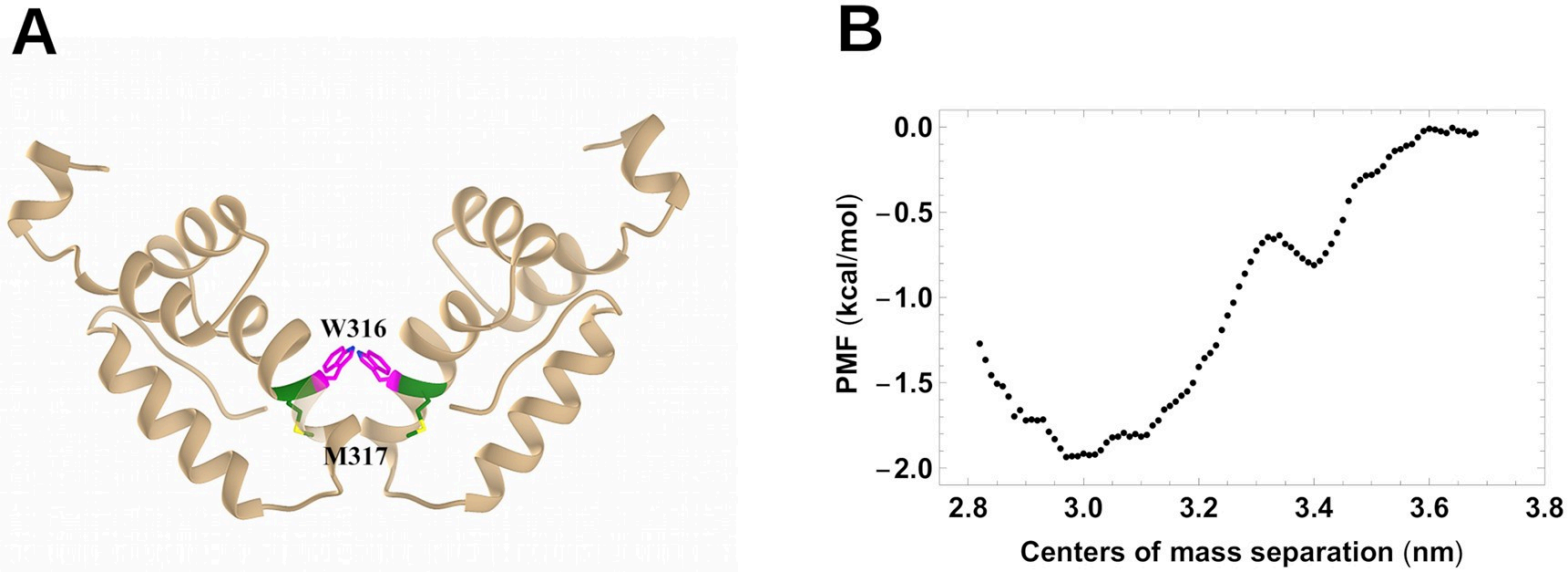
CA_ctd_ two-fold contact in the immature capsid and associated PMF. A:Two CA_ctd_ subdomains (tan) remained in contact after a 80 ns simulation, bound by their corresponding nonpolar residues W316 (violet) and M317 (green). B: Potential of Mean Force between the center of mass of the two CA_ctd_ subdomains, obtained by umbrella sampling MD simulation (black points).

In order to place the intermolecular MA/CA_ctd_ bond in context, we compared it with the intermolecular bond between two Gag proteins that are part of the Gag lattice of the immature virion. The cryo-electron-microscopy structures of the immature Gag lattice of Refs. [14, 15] indicate that the assembly-critical residues W316 and M317 of one CA_ctd_ domain form a homo-dimeric hydrophobic contact with the W316 and M317 domains of the CA_ctd_ domain of an adjacent Gag protein. We computed the PMF of two CA_ctd_ domains with the starting state of the simulation produced by excising a pair of CA_ctd_ domains from across the two-fold symmetry sites of the hexagonal lattice. The pair first was thermally equilibrated by an 80 ns MD simulation (see Fig 6A), during which the bond remained intact, followed by an umbrella-sampling measurement of the PMF.

The PMF of the CA_ctd_ dimer is shown in Fig 6B. It has a potential well with a depth Δ*V*_*CC*_ of about 1.9 kcal/mol (∼ 3.2 *k*_*B*_*T*) and a range of 0.6 nm. Unlike the MA/CA_ctd_ PMF, there is no extended region with constant slope. The CA_ctd_/CA_ctd_ binding free energy Δ*V*_*CC*_ is about three times smaller than the MA/CA_ctd_ binding free energy. Thus, the mainly-electrostatic MA/CA_ctd_ interaction is apparently strong enough to compete with these hydrophobic CA_ctd_/CA_ctd_ interactions that contribute importantly to the immature-virion Gag lattice stability.

## Discussion and conclusions

Our simulations support the proposal that the Gag protein has a bound state (C-Gag) stabilized by the interaction between its MA and CA_ctd_ domains and allowed sterically by the 30 amino acid-long, unstructured and flexible linker between the MA and CA domains. Against our expectations, the simulations indicated that this bound state is primarily stabilized by electrostatics with dipolar interactions between residues (Q63,T70 of MA and T348, Q351 of CA_ctd_) and monopolar interactions between the charged residues R43 of MA and E344, E345 of CA_ctd_. The strength of this intra-Gag interaction, measured by the depth of its associated potential of mean force, is surprisingly strong: 6.8 kcal/mol ∼ 11*k*_*B*_*T*. For comparison, the major interaction known to stabilize Gag-Gag dimerization within the immature lattice (via hydrophobic sites centered at W316, W317 of CA_ctd_) is much weaker, we determined that its PMF depth is only 2.0 kcal/mol ∼ 3.3*k*_*B*_*T*.

It already is well appreciated that electrostatic interactions play an important role for the state of the Gag protein in general and for RNA selection in particular [20, 21]. MA has a non-specific affinity for RNA molecules [46] and the plasma membrane [47] believed to be due, at least in part, to the large number of positively charged residues of MA. Separately, the NC domain appears to bind to RNA by a combination of non-specific electrostatic interactions that involve positively charged residues of NC and specific hydrophobic interactions [48–51]. The MA/CA_ctd_ bound state we are reporting on is mostly stabilized by dipolar interactions. Because binding between dipolar molecules is weaker than that between molecules with opposite monopole charges the total MA/CA_ctd_ binding energy of 6.8 kcal/mol necessitates multiple dipolar contacts, which we indeed found to be the case.

With such a strong interaction stabilizing the C-Gag state, how can Gag transit to its extended state forming the immature Gag lattice? Another major contributor to the stability of that lattice comes from the six-helix bundle formed by attraction of SP1 regions [52]. This involves a transition of the SP1 region and the adjacent C-terminal of CA_ctd_ from its unstructured coil state in monomeric Gag to an *α*-helical state of the same region in the bundle state [11, 12, 53]. Experimental evidence by Rein *et al*. [12, 54], who observed that critical mutations in SP1 destabilizing the six-helix binding *in vitro* also lead to defects of the assembly in cells of the immature virion comparable to those produced by WM Gag dimerization mutation, allows one to estimate that the strength of the six-helix bundle interaction is of the same order of 2-4 kcal/mol. In addition, it was recently discovered that the six-helix-bundle of SP1 is significantly stabilized by binding of the inositol hexaphosphate (IP6) molecule in between the six helices [52] and that such IP6 bound molecules are present in the immature Gag lattice in cells. Increase in the immature-like *in vitro* particle assembly upon addition of IP6 suggests that the contribution of IP6 binding to immature lattice assembly is comparable to the strength of SP1-SP1 contacts without IP6 [52, 55]. Taken together, the SP1-SP1+IP6 and WM-WM contacts would appear to be sufficiently strong to compete with the monomeric C-Gag conformation and drive the immature virion assembly.

The intra-Gag binding site identified in this work in CA_ctd_ is spatially distant from the WM dimerization site, but it is close (both spatially and within the sequence) to the SP1 junction region (residues 356-373). The two lysine residues (290, 359) known to coordinate IP6 [52] are also very close, therefore we expect formation of the six-helix bundle of SP1 not only to compete energetically but also sterically with the C-Gag conformation.

The MA portion of the MA/CA_ctd_ binding site is located on the opposite face from the Highly Basic Region (HBR) involving eight cationic residues [56], see Fig 2A. HBR is known to be the site of competitive binding of MA to either the plasma membrane or RNA [46]; hence, we do not expect MA/CA_ctd_ binding to interfere with either of these MA functions, which is consistent with the known ability of MA to bind to the plasma membrane either compact (as a monomer) [57] or extended (as in immature virions). Interestingly, the MA portion of the MA/CA_ctd_ binding site coincides with the MA site involved in its trimerization on plasma membrane [58]. The MA trimerization interaction is known to be fairly weak, not contributing much to the stability of the Gag immature lattice, being more important for the envelope insertion rather that Gag-Gag interactions [58]. However, involvement of the MA trimerization site in the strong intra-Gag interaction would certainly interfere sterically with the formation of immature-like lattice of MA domains between plasma membrane-bound C-Gag molecules.

Also of interest is the degree of sequence conservation of the newly found intra-Gag site, since such a measure correlates both with its functional importance [59] and suitability as an antiviral drug binding site [60]. Also, because the MA/CA_ctd_ bond is held together by a significant number of weak polar contacts that collectively determine the binding free energy, we should expect that the extended polar MA/CA_ctd_ binding interface is conserved in terms of mutations. An extensive analysis of Gag sequence conservation [59] found that, among almost 11,000 sequences over multiple strains of eight HIV-1 subtypes, the average sequence variability for the entire Gag protein is 43.6%, with its CA domain being the least variable (29.4)%. First, we notice that all six residues in CA_ctd_ involved with the intra-Gag contact are highly conserved: five of them (R294, E344, E345, M347, Q351) are fully conserved and the remaining one (T348) has a very low variability (1.6%). Next, five out of these six residues match known sites of anti-HIV-1 drug binding [60]: of the eight CA_ctd_ residues that the small peptide CAI –an inhibitor of viral assembly– binds to, three of them (V297, L343 and M347) coincide with or are proximal to every CA_ctd_ residue in the MA/CA_ctd_ binding interface, with the exception of Q351 [61]. Finally, residue R294 is part of the *major homology region* (MHR), a group of 19 consecutive residues (285-304) known to be almost completely conserved not only across various HIV-1 strains, but also among other retroviruses and retrotransposons. While the function of MHR is still unclear [16], deletion of MHR hinders Gag assembly and viral infectivity.

Taken together, these findings imply high functional significance for the intra-Gag binding site in CA_ctd_ and we suggest that this function would be stabilization of the compact Gag state and thus, indirectly, participation in gRNA selective packaging. In this regard, extensive alanine scanning mutations of the HIV-1 Gag surface was performed by Sundquist and co-authors [62] in order to identify Gag residues essential for its immature and mature assembly and infectivity. A number of mutations at the base of CA_ctd_ were identified that affected infectivity. However, the residues found in our work to participate in the intra-Gag binding to MA were not tested, except for the E344A mutation that was shown to lead to moderate infectivity defect, without assembly reduction. This result is consistent with our prediction of the intra-Gag contacts being important for gRNA packaging, but not for the immature-lattice Gag-Gag contacts. A revisiting of these mutational studies is warranted by our computational findings.

Our simulations regarding increasing alanine substitutions in the MA binding interface point to a gradual deterioration of its binding effectivity as the number of such point mutations increases. We consider probable that, while the residues that we focused on based on consensus of the Protein-Protein Interaction algorithms are the most easily identifiable contributors to the binding interface, other contacts that were listed by a majority of algorithms play a role too (see S1 Table in Supplementary Information). For one thing, these other contacts could provide some non-specific attraction to keep the MA and CA_ctd_ domains in contact, hence providing a larger basin of attraction for the strongest contact to be found. This would amount to a two-step process for a MA/CA_ctd_ dimer searching for the optimal binding configuration: first, non-specific attraction would bring the two domains into close proximity regardless of orientation, then the search could proceed orientationally until the strongest bond is found. We are currently quantifying this effect using simplified coarse-grain models that will be presented in a separate communication.

Our molecular dynamics simulations disproved our second hypothesis concerning a key role for the assembly-critical hydrophobic residues W316, M317 of the CA_ctd_ domain. These residues remain fully exposed to the aqueous environment. This has the interesting consequence that two C-Gag proteins should be able to dimerize through hydrophobic interactions and possibly form VLPs. The C-Gag bound state is sterically quite different from the extended conformation of Gag proteins that are part of a hexagonal immature lattice. Thus, particles assembled of C-Gag should be quite different from the immature-like VLPs. In-vitro self-assembly studies that involve mixing RNA and Gag in physiological salt buffer, report formation of ≃30 nm diameter particles [63] in contrast to the ≃130 nm diameter virion-like VLPs [64]. We propose that these small-sized VLPs, which are relatively unstable, are composed of C-Gags stabilized via interaction between the exposed CA_ctd_ hydrophobic residues W316 and M317.

According to the physics of aqueous electrostatics, binding between oppositely charged macroions should be weakened by the addition of polyvalent ions while addition of monovalent salt should have the same effect. The addition of negatively charged tRNA [21] and of IP6 groups [65] both increase the binding specificity of Gag for gRNA. Separately, the addition of the IP6 molecule stabilizes normal-sized VLPs over the small-sized VLPs [64]. A natural interpretation would be that the association of the negative polyions with the positive MA residues weakens the bound state but this is not so obvious because of the presence of the large number of MA positive residues that lie outside the binding interface: binding of IP6 or tRNA to MA would be expected to involve mainly these positive MA residues. However, some of the positive MA residues are immediately adjacent to the polar residues of the binding interface so the MA/CA_ctd_ bound state still could be disrupted by short-range allosteric coupling between the charged and polar residues when tRNA or IP6 binds to the positively charged MA residues. Related to this, if the MA domain would be more flexible in the extended state than in the bound state, then this also could favor disruption of the bound state because that flexibility would facilitate the binding of the tRNA and IP6 groups to the positively charged residues. These possibilities can all be investigated by MD simulations and we plan to do this. Finally, long-range allosteric interactions between the NC and MA/CA parts of Gag also could play a role in terms of the interaction of MA with tRNA and IP6. Checking this requires more complex simulations of the whole Gag proteins.

The effect of increased monovalent salt concentration on the RNA binding specificity of Gag has been investigated. [20]. At physiological NaCl concentration ∼150 mM, Gag binds with similar strength to non-specific RNA and to gRNA. Increasing the NaCl concentration weakens the Gag interaction with non-specific RNAs, while binding to RNA containing the *Ψ* packaging signal remains rather strong. Gag binding non-specific RNA shows an effective charge of +10 (including contributions from both NC and MA) and negligible non-electrostatic Gag/RNA interactions. In contrast, a smaller effective charge (about +5) for Gag binding to *Ψ* RNA, along with considerable non-electrostatic interactions, leading to significant binding in high salt was reported. It was hypothesized that, between 50 and 500 mM of NaCl, Gag binds *Ψ* RNA in the E-Gag state, while it binds non-specific RNA in the C-Gag state.

Following this proposal, one can expect that the intra-Gag contact found in the present work leads to some rigidification of C-Gag, directly in MA and (as suggested by previous simulations [18]), indirectly, in NC. This would imply some loss of ability to optimize non-electrostatic contacts with RNA, such as stacking interactions between the aromatic residues of the zinc fingers of NC and unpaired G bases of RNA. By contrast, in the E-Gag conformation the NC and MA domains would be free to move and optimize their stacking interactions with RNA. Since specific contacts of NC with *Ψ* RNA are much stronger than with non-specific RNA, this could stabilize E-Gag over C-Gag upon *Ψ* RNA binding, but not upon non-specific RNA binding. As only the extended state is able to initiate virion assembly, this may provide a mechanism of gRNA packaging selectivity as proposed in Ref. [20, 66].

A second study [21] confirmed that increased salt concentration enhances gRNA binding specificity and that competition between non-specific electrostatic interactions with non-specific hydrophobic interactions play an important role. There is a surprising dependence on salt concentration of the dissociation constant of the binding of Gag to RNA: it is relatively independent of salt concentration at low values but, beyond a threshold concentration, it rises sharply at higher values. In contrast, one would expect on the basis of aqueous electrostatics that increased salt concentration should immediately weaken the generic electrostatic interactions and that only when non-electrostatic interactions dominate should the dissociation constant be independent of salt concentration. The authors of [21] interpret these results in terms of a salt-induced conformational change of Gag and propose the presence of a compact state of Gag stabilized by electrostatic interactions. Our simulations revealing a strongly-bound MA/CA_ctd_ conformation are consistent with the experimental results pointing to the presence of a compact Gag state, but it is clear that MD simulations of this bound state should be undertaken at different salt concentrations and compared with simulations of the NC-RNA interaction before drawing further conclusions.

## Supporting information

S1 Appendix Root-mean-square deviation of atomic coordinates among several CA_ctd_ PDB structures. (PDF)

S2 Appendix Brief description of the umbrella sampling method. (PDF)

S1 Fig Comparison of several CA_ctd_ PDB structures. (TIF)

S1 Table Lists of residues in the MA/CA_ctd_ binding interface given by seven protein-protein interaction algorithms. (XLSX)
