# Supplementary material for: Specific inter-domain interactions stabilize a compact HIV-1 Gag conformation
Source: PLoS One. 2019 Aug 22;14(8):e0221256. doi: 10.1371/journal.pone.0221256 (PMC6705756; doi:10.1371/journal.pone.0221256)
Supplement: S1 Appendix — (PDF) [file pone.0221256.s001.pdf]

S1 Appendix: Root-mean-square deviation of atomic coordinates among several CA_{ctd} PDB structures

We used the UCSF Chimera program version 1.12 [1] to visualize and generate alignments for the different structures of CA_{ctd} considered in this work, with the option "match align with iteration". This option generates an alignment sequence from a structural superposition of proteins, using their spatial proximities. We also used the "iterate by pruning" procedure, which refines the structure alignment by progressive elimination of the worst aligned pair in each iteration. The program returns two root-mean-square deviation (RMSD) values: the first one corresponds to the pruned calculation and the second one considering all atoms in each residue. We report in the main text the second value as the RMSD for each pair of structures considered and quote the full program output below.

```
Matched 4USN-A.pdb, chain A  vs 5l93-A.pdb, chain A
RMSD 0.201  with 9 pruned atom pairs; RMSD 1.446 across all 68 atom pairs

Matched 4USN-A.pdb, chain A vs 5I4T-G.pdb, chain G
RMSD 0.181  with 8 pruned atom pairs; RMSD 1.272 across all 68 atom pairs

Matched    4USN-A.pdb, chain A vs 6N3J-A.pdb, chain A
RMSD  0.157  with 5 pruned atom pairs; RMSD 1.413 across all 68 atom pairs
```

References

1. Pettersen EF, Goddard TD, Huang CC, Couch GS, Greenblatt DM, Meng EC, et al. UCSF Chimera–a visualization system for exploratory research and analysis. J Comput Chem. 2004;25(13):1605–12.
