# Supplementary material for: Specific inter-domain interactions stabilize a compact HIV-1 Gag conformation
Source: PLoS One. 2019 Aug 22;14(8):e0221256. doi: 10.1371/journal.pone.0221256 (PMC6705756; doi:10.1371/journal.pone.0221256)
Supplement: S2 Appendix — (PDF) [file pone.0221256.s002.pdf]

S2 Appendix: Brief description of the umbrella sampling method

Umbrella sampling allows one to estimate the difference in free energy associated with changes in a collective variable [1, 2]. It works by introducing a collection of simulations, each one applying a soft restraint (such as a harmonic potential, $W(X - X_k)$) to bias the variable towards a particular value X_k. In our case, from each simulation we collected a biased distribution of values of the COM separation X between two proteins, in the form of a sequence of overlapping histograms $P_k(X)$ over succesive windows. The global unbiased distribution $P(X)$ and associated free energy

$$A(X) = -k_{\mathrm{B}} T \ln P(X), \tag{1}$$

are obtained by linear superposition of unbiased distribution estimates from each window,

$$\overline{P}_k(X) = e^{\beta A_k} e^{\beta W(X - X_k)} P_k(X). \tag{2}$$

The superposition coefficients are optimized to minimize the statistical error of the global distribution $P(X)$. This optimization is carried out numerically by an interative procedure: a commonly used implementation is that known as the weighted histogram analysis method (WHAM) [3]. The potential of mean force $PMF(X)$ can be computed as the free energy difference

$$PMF(X) = A(X) - A(X_0) \tag{3}$$

associated with the point of interest X and some reference X_0. In practice, we may choose any reference value X_0 sufficiently large to ensure that the molecules in the complex do not interact signficantly, a situation reflected by the free energy "flattening out" and becoming independent of separation.

References

1. Frenkel D, Smit B. Understanding Molecular Simulation: From Algorithms to Applications, 2nd Ed. Academic Press; 2002.

2. Tuckerman M. Statistical Mechanics: Theory and Molecular Simulation. Oxford Universiyt Press; 2005.

3. Kumar S, Bouzida D, Swendsen RH, Kollman PA, Rosenberg JM. The weighted histogram analysis method for free-energy calculations on biomolecules. I. The method. J Comput Chem. 1992;13:1011–1021.
