# Supplementary figures and images for: Specific inter-domain interactions stabilize a compact HIV-1 Gag conformation
Source: PLoS One. 2019 Aug 22;14(8):e0221256. doi: 10.1371/journal.pone.0221256 (PMC6705756; doi:10.1371/journal.pone.0221256)

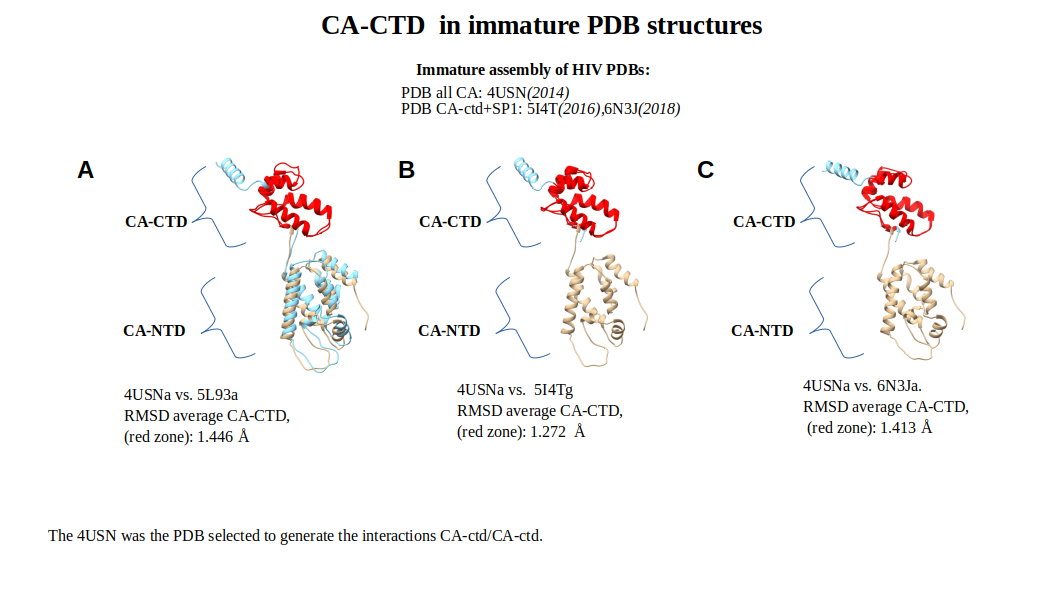

Supplement: S1 Fig — (TIF) [file pone.0221256.s003.tif]
